# In this issue: Volume 114, Issue 1, January 2023

**DOI:** 10.1111/cas.15404

**Published:** 2023-01-02

## Nucleic acid–triggered tumoral immunity propagates ph‐selective therapeutic antibodies through tumor‐driven epitope spreading


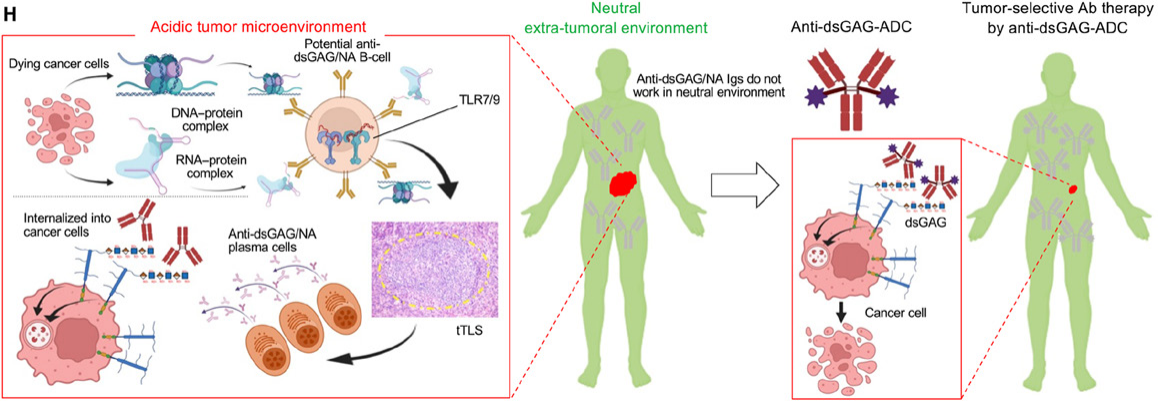


Innate or natural immunity and acquired immunity are the two major modes of defense in the human body. Acquired immunity is further classified into cell‐mediated immunity, driven by T cells, and humoral immunity, driven by B cells. B cells produce specific antibodies against foreign antigens, such as pathogens and even cancers. In fact, studies show an abundance of B cells in the tumor microenvironment (TME), i.e., the milieu surrounding a tumor. However, their function and the antigens they respond to in the TME are not well‐known. This precludes an overall understanding of how antibodies fight cancer, which could have immense therapeutic potential.

In this study, Furuya et al. set out to find answers to this pressing question. Using samples from 102 patients with gastric cancer and 27 patients with pancreatic cancer, they identified the antibodies (i.e., immunoglobulins) predominantly present in the TME.

Contrary to their expectation, they discovered low immunoglobulin diversity in the TME. Around one‐third (10/30) of the dominant immunoglobulins they identified exhibited dual‐targeting effects against two types of molecules. These were densely sulfated glycosaminoglycans (dsGAGs), which are often expressed on the surface of cancer cells, and nucleic acids (NAs), key cellular components that leak out from dead cells and are abundant in the TME. Together, these findings indicated that B cells in the TME respond to and target specific tumor‐specific signatures in order to fight cancer.

Another key signature of tumors and the TME is the low (acidic) pH. Hence, the team wished to understand how anti‐dsGAG/NA antibodies emerge and multiply in the acidic TME. Through tests of reconstructed antibodies and cell culture experiments, they found that the body's innate anti‐NA immunity triggers the emergence of a highly dominant milieu of anti‐dsGAG/NA antibodies.

Notably, these antibodies are pH‐selective, i.e., their activity is enhanced under acidic conditions. Thus, in the acidic TME, these antibodies are quickly internalized by cancer cells. The interaction between innate and acquired immunity affects the emergence and maturation of anti‐dsGAG/NA antibodies in the TME.

Finally, to leverage the therapeutic potential of their findings, the team developed antibody‐drug conjugates (ADCs) using anti‐dsGAG/NA antibodies. Analysis of tumor tissues suggested that the ADCs could target a wide range of human malignancies, including various epithelial cancers, even though the original anti‐dsGAG/NA antibodies were derived from gastric cancer samples.

These findings highlighted the potential applications of these antibodies and its drug conjugates for the universal treatment of a wide spectrum of malignancies.

https://onlinelibrary.wiley.com/doi/full/10.1111/cas.15596

## Novel hkdr mouse model depicts the antiangiogenesis and apoptosis‐promoting effects of neutralizing antibodies targeting vascular endothelial growth factor receptor 2


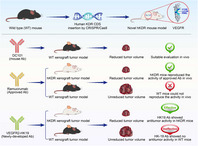


Angiogenesis, or the formation of new blood vessels, can increase blood supply to tumors. This, in turn, leads to the rapid progression of cancer. Blocking the growth of new blood vessels around the tumor mass is thus an effective strategy for tumor growth suppression. An important molecular target in this regard is the vascular endothelial growth factor receptor 2 (VEGFR2/ KDR). By binding VEGFR2 on the surface of endothelial cells, therapeutic agents like ramucirumab block downstream activities responsible for cell proliferation and migration.

The current state of research on this topic is limited by the fact that the VEGFR2 protein sequence is quite different for mice and humans, making it difficult to test hypotheses and translate research. To address this gap, in a recent study, researchers in China used the CRISPR/Cas9 genome editing technique to modify the KDR sequence in the corresponding mouse protein to make it similar to human VEGFR2. The resultant humanized or hKDR mice allow the effective evaluation of therapeutics targeting VEGFR2/KDR.

Additionally, the researchers created a new monoclonal antibody, VEGFR2‐HK19. Monoclonal antibodies are very specific in their binding and can allow efficient targeting of molecules. The researchers found that VEGFR2‐HK19 inhibits tumor cell proliferation by inducing apoptosis or cell death. When they compared VEGFR‐HK19 and ramucirumab, they found that the former had a higher affinity and superior antiproliferation activity.

Thus, through their study, the researchers have established a humanized KDR mouse model that can help test the efficiency of certain anticancer therapeutics. Their results also suggest that VEGFR2 is a possible tumor marker for metastasis. In addition, they were able to create a monoclonal antibody that can be a potentially more effective therapeutic than the existing treatment option, ramucirumab. The results of this study will provide a much‐needed platform for future drug research and development.

https://onlinelibrary.wiley.com/doi/full/10.1111/CAS.15594

## Hypoxia‐induced cxc chemokine ligand 14 expression drives protumorigenic effects through activation of insulin‐like growth factor‐1 receptor signaling in glioblastoma


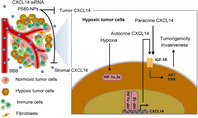


Glioblastomas are a type of aggressive cancer of the brain or spinal cord. They are known to turn malignant when oxygen levels within the tumor are low, a condition known as a hypoxic microenvironment. Studying the altered expression of genes in hypoxic tumors can reveal potential molecular targets for the treatment of glioblastoma.

One such molecule is the CXC chemokine ligand 14 (CXCL14), which has elevated expression in hypoxic tumors when compared with normal brain cells. This elevated expression is associated with poor overall survival of patients. However, the mechanisms underlying high CXCL14 expression in hypoxia and the subsequent events leading to poor patient outcomes remain unclear.

To address this gap, researchers in Taiwan performed in vivo and in vitro studies and found that the increase in CXCL14 levels was triggered by hypoxia‐inducible factors. They also demonstrated that high expression of CXCL14 in glioblastomas increased the activation of insulin‐like growth factor‐1 receptor (IGF‐1R), an important component of cell signaling pathway, which leads to the greater growth and invasiveness of tumors.

The researchers next used small interfering RNA (siRNA) nanoparticles to suppress the expression of CXCL14 in live tumor cells. Administration of siRNA nanoparticles slowed cancer progression and could improve survival of patient‐derived tumor tissues grafted onto live mice. What's more, coating these nanoparticles with the nonionic emulsifier polysorbate 80 (PS80) improved the tumor suppression efficacy considerably. These findings suggest that CXCL14 siRNA nanoparticles can be used as a potential drug for glioblastoma treatment.

This study sheds light on the connection between hypoxia and tumor growth in glioblastoma via CXCL14. The researchers suggest that besides its role in activating IGF‐1R and its downstream biochemical mediators, CXCL14 may also play a role in proliferating fibroblasts or endothelial cells as well as regulating infiltration of immune cells like dendritic cells, natural killer cells, and T cells. Together, these may contribute to the growth and proliferation of glioblastomas. Further research is needed to elucidate these pathways. Nonetheless, a better understanding of CXCL14's mechanism of action as provided by this study opens avenues for the development of novel drugs, such as CXCL14 siRNA nanoparticles, for the treatment of glioblastomas.

https://onlinelibrary.wiley.com/doi/full/10.1111/CAS.15587

